# Safety and therapeutic efficacy of artemether-lumefantrine in the treatment of uncomplicated *Plasmodium falciparum* malaria at Shecha health centre, Arba Minch, Ethiopia

**DOI:** 10.1186/s12936-022-04436-8

**Published:** 2023-01-07

**Authors:** Kale Gubae, Hussein Mohammed, Heven Sime, Henok Hailgiorgis, Anteneh Kassahun Mare, Bokretsion Gidey, Mebrahtom Haile, Gudissa Assefa, Worku Bekele, Geremew Tasew, Solomon Mequanente Abay, Ashenafi Assefa

**Affiliations:** 1 Department of Pharmacy, College of Health Sciences, Debre Markos University, Debre Markos, Ethiopia; 2 Department of Pharmacology and Clinical Pharmacy, School of Pharmacy, College of Health Sciences, Addis Ababa University, Addis Ababa, Ethiopia; 3 Malaria and Other Parasitological and Entomological Research Team, Bacterial, Parasitic and Zoonotic Diseases Research Directorate, Ethiopian Public Health Institute, Addis Ababa, Ethiopia; 4 Ethiopian Ministry of Health, Addis Ababa, Ethiopia; 5 World Health Organization, Addis Ababa, Ethiopia; 6 Institute of Infectious Disease and Global Health, University of North Carolina at Chapel Hill, Chapel Hill, USA

**Keywords:** Therapeutic efficacy, Artemether-lumefantrine, Uncomplicated malaria, Ethiopia

## Abstract

**Background:**

In 2004, Ethiopia adopted artemether-lumefantrine (AL, Coartem^®^) as first-line treatment for the management of uncomplicated *Plasmodium falciparum* malaria. Continuous monitoring of AL therapeutic efficacy is crucial in Ethiopia, as per the World Health Organization (WHO) recommendation. This study aimed to assess the therapeutic efficacy of AL in the treatment of uncomplicated *P. falciparum* infection.

**Methods:**

A 28 day onearm, prospective evaluation of the clinical and parasitological response to AL was conducted at Shecha Health Centre, Arba Minch town, Southern Ethiopia. Patients were treated with six-dose regimen of AL over three days and monitored for 28 days with clinical and laboratory assessments. Participant recruitment and outcome classification was done in accordance with the 2009 WHO methods for surveillance of anti-malarial drug efficacy guidelines.

**Results:**

A total of 88 study participants were enrolled and 69 of them completed the study with adequate clinical and parasitological response. Two late parasitological failures were observed, of which one was classified as a recrudescence by polymerase chain reaction (PCR). The PCRcorrected cure rate was 98.6% (95% CI 92.3–100). AL demonstrated a rapid parasite and fever clearance with no parasitaemia on day 2 and febrile cases on day 3. Gametocyte clearance was complete by day three. No serious adverse events were reported during the 28 days follow-up.

**Conclusion:**

The study demonstrated high therapeutic efficacy and good safety profile of AL. This suggests the continuation of AL as the first-line drug for the treatment of uncomplicated *P. falciparum* malaria in Ethiopia. Periodic therapeutic efficacy studies and monitoring of markers of resistance are recommended for early detection of resistant parasites.

**Supplementary Information:**

The online version contains supplementary material available at 10.1186/s12936-022-04436-8.

## Background

Currently, artemisinin-based combination therapy (ACT) is the most effective and widely used treatment for uncomplicated malaria caused by *Plasmodium falciparum* [1]. Expanded access to ACT in malaria-endemic countries has helped to reduce the worldwide burden of this illness significantly over the last 15 years [2]. However, *P. falciparum*, which still remains responsible for the great majority of malaria deaths globally, has shown resistance to ACT [3]. Partial resistance to artemisinin, characterized by delayed parasite clearance, is now widespread in the Greater Mekong Subregion of Southeast Asia. There is no evidence of ACT resistance spreading to other parts of the world from this region, but an eventual spread of resistance to other parts of the world cannot be overlooked. In Africa, despite the detection of PfK13 mutations, ACT remains efficacious [4]. Emergence of artemisinin resistance in this region, particularly to artemether–lumefantrine (AL), the most widely used anti-malarial, is a threat to the achievements gained so far in malaria control and elimination endeavours.

To combat the threatening ACT resistance, periodic therapeutic efficacy studies (TES) in selected sites form the basis of surveillance system for monitoring changes in drug efficacy over time. The World Health Organization (WHO) recommends a change in treatment policy when treatment failure rate is more than 10% during the observation period [4].

Ethiopia introduced AL as the first-line treatment for uncomplicated *P. falciparum* malaria in 2004 [5]. At the time of its introduction the therapeutic efficacy of AL was 99.1% [6]. Since then, a number of studies have been conducted at different sentinel sites, with a recent study indicating a 96% PCR-corrected cure rate of AL [7]. According to the updated WHO recommendation [8], this did not necessitate a policy change. However, surveillance of anti-malarial therapeutic efficacy and resistance is required to detect changing patterns of parasite susceptibility and make timely revisions to national and global policies. In this regard, this TES aimed to determine the current efficacy of AL in Arba Minch town, Southern Ethiopia, thereby assisting the Ministry of Health in the preparation of evidence-based treatment strategies and policies.

## Methods

### Study design and duration

This surveillance study was a one-arm prospective evaluation of clinical and parasitological responses to the treatment of AL for uncomplicated malaria. The study was conducted between March 2021 and January 2022.

### Study setting

This study was conducted in Shecha health centre, Arba Minch town. Arba Minch town is administratively located in Gamo Gofa zone of the Southern Nations, Nationalities and Peoples’ Region of Ethiopia. Arba Minch town, is located at about 500 km South of Addis Ababa, the capital city of Ethiopia. At an altitude of 1200–1400 m above sea level, the town has an average annual rain fall of 800–1000 mm and an average annual temperature of 29.0 °C. The administration of Arba Minch town is divided into four kifle-ketemas (sub-towns) -Shecha, Sikella, Abaya and Nechsar. The four kifle-ketemas totally comprise 11 kebeles, all of which are malarious areas.

### Study population

The population consisted of outpatients over six months of age, with uncomplicated *P. falciparum* malaria attending the study health centre.

### Sample size calculation

According to the working WHO protocol for anti-malarial TES, a minimum of 73 participants should be recruited to detect an expected failure rate of 5% at a confidence level of 95% and an estimate precision of 5% [8]. To account for follow-up and withdrawal losses, an additional 20% was added to the total. Following this calculation, 88 study participants were recruited for the study.

### Eligibility criteria

Inclusion criteria were patient of six months or older, having a microscopically positive *P. falciparum* mono-infection, having an asexual parasitaemia level of 1000 to 200,000/µl, having an axillary temperature of 37.5 °C or higher at presentation or having a history of fever within the previous 24 h, being able and willing to comply with the study protocol and study visit schedule for the duration of the study, and being willing to sign an informed consent or assent (children under 12 years old) form.

The following exclusion criteria were considered for the study. General danger signs in children under the age of 5 years or signs of severe falciparum malaria; weight under 5 kg; haemoglobin < 8 g/dl; severe malnutrition; the presence of febrile conditions caused by diseases other than malaria or other known underlying chronic or severe diseases; regular medication, which may impair anti-malarial pharmacokinetics; a history of hypersensitivity reactions or contraindications to AL; pregnancy or breastfeeding; being unable to or unwilling to take contraceptives (for women of child-bearing age).

### Sample collection

Thick and thin blood films were obtained and examined at screening on day 0 to confirm adherence to the inclusion and exclusion criteria. Thick blood films were also examined on days 2, 3, 7, 14, 21, 28, or any other day when the patient returned spontaneously, and parasitological reassessment was required. Specimens were labelled anonymously (study number and day of follow-up). Two to three drops of blood samples were collected on Whatman 903 filter paper during screening and each time treatment failures were encountered. Specimens were labeled anonymously (study number, day of follow-up), dried, kept in individual plastic bags with desiccant pouches and protected from light, humidity, and extreme temperature.

### Microscopic analysis

Three blood slides per patient were obtained at enrolment: two thick blood films and one thin blood film. One thick blood film was then stained rapidly (10% Giemsa 15 min) for initial screening, while the others were retained. When the patient was subsequently enrolled, the second blood film was stained slowly (3% Giemsa for 45 min). This was used to calculate the parasite density, by counting the number of asexual parasites in a set number of white blood cells (typically 200) with a hand tally counter. The same technique was used to establish the parasite count on each subsequent blood film. A blood film was considered negative when examination of 1000 white blood cells (WBCs) showed no asexual parasites. The presence of gametocytes on an enrolment or follow-up slide was recorded. In addition, 100 fields of the second thick film were examined to exclude mixed infections; in case of any doubt, the thin film was examined. Parasite density was calculated as follows:

$${\text{Parasite density}}\, (\text{Per}\; \mu l) = ({{\text{parasites counted}} \mathord{\left/ {\vphantom {{\text{parasites counted}} {\text{WBCs counted}}}} \right. \kern-0pt} {\text{WBCs counted}}})\;8000$$

As a quality control measure, all slides were examined by two senior WHO-certified microscopists and during discrepancy a third microscopist read the result and the agreed results were final.

### Haemoglobin measurement

Finger-pick blood samples were used to measure haemoglobin on day 0, day 14 and day 28. Haemoglobin was calculated by taking one third of the hematocrit level.

### Treatment and followup

AL (20 mg/120 mg) [Batch No.: (10): HWE110049; Mfg: (11), 01/2020; Exp: (17):12/2021] manufactured by Ipca Laboratories Ltd (India) was obtained from the WHO Addis Ababa office. Participants were treated twice daily for three consecutive days with the standard six-dose regimen of AL (Additional file 1: Table S1). Drug dosage was determined according to the patient’s body weight. The first dose of the medicine was administered under the supervision of a well-trained clinician. The study patients were observed for 30 min after medicine administration for adverse events or vomiting. Any patient who vomited during this observation period were re-treated with the same dose of medicine and observed for an additional 30 min. The first doses of day-1 (2 d of treatment) and day-2 (3 d of treatment) were directly observed by the study staff, while evening doses were given each day to patients or guardians to be administered at home”. Patients were verbally monitored if they encountered any problem in taking the second dose and confirm taking of the drug. On each day, the first doses for children were given with milk biscuits. All participants were also advised to take the evening doses with food. Thereafter, patients were required to undergo regular clinical and parasitological reassessment as detailed above. Patients or guardians were advised to return on any day during the follow-up period if symptoms returned and not to wait for the next scheduled visit day.

### Patient withdrawal

The following general criteria were set forth to classify patients as withdrawn: withdrawal of consent; failure to complete treatment (due to: persistent vomiting of the treatment, failure to attend the scheduled visits during the first three days, or serious adverse events necessitating termination of treatment before the full course is completed); enrolment violation (i.e. severe malaria on day 0, erroneous inclusion of a patient who does not meet the inclusion criteria); voluntary protocol violation (i.e. self- or third-party administration of anti-malarial drug or antibiotics with anti-malarial activity); involuntary protocol violation (i.e. occurrence during follow-up of concomitant disease that would interfere with a clear classification of the treatment outcome, detection of a mono-infection with another malaria species during follow-up, or misclassification of a patient due to a laboratory error (parasitaemia), leading to administration of rescue treatment).

### Classification of treatment outcomes

Treatment outcome of patients were classified according to the WHO guideline [8]. On the basis of an assessment of the parasitological and clinical outcome the protocol classifies treatment outcomes as early treatment failure (ETF), late clinical failure (LCF), late parasitological failure (LPF) or an adequate clinical and parasitological response (ACPR).

#### Early treatment failure

Development of danger signs or severe malaria on day 1, 2 or 3, in the presence of parasitaemia; parasitaemia on day 2 higher than on day 0, irrespective of axillary temperature; parasitaemia on day 3 with axillary temperature ≥ 37.5 °C; and parasitaemia on day 3 ≥ 25% of count on day 0.

#### Late clinical failure

Development of danger signs or severe malaria in the presence of parasitaemia on any day between day 4 and day 28 in patients who did not previously meet any of the criteria of early treatment failure; and presence of parasitaemia on any day between day 4 and day 28 with axillary temperature ≥ 37.5 °C in patients who did not previously meet any of the criteria of early treatment failure.

#### Late parasitological failure

Presence of parasitaemia on any day between day 7 and day 28 with axillary temperature < 37.5 °C in patients who did not previously meet any of the criteria of early treatment failure or late clinical failure.

#### Adequate clinical and parasitological response

Absence of parasitaemia on day 28, irrespective of axillary temperature, in patients who did not previously meet any of the criteria of ETF, LCF or LPF.

### Safety endpoint

Safety was assessed by recording the nature and incidence of adverse events. Adverse events were assessed by direct interview. All patients were consistently asked about prior symptoms as well as new symptoms that had appeared since their last appointment. The incidence of any adverse event, independent of its relationship to the study drug, was used as a safety end-point. An adverse event was defined as any unfavourable, unintended sign, symptom, syndrome or disease that develops or worsens with the use of the study drug, regardless of whether it is related to the medicinal product.

### Molecular genotyping

Dried blood spots were obtained for PCR analysis on day 0 and on days of treatment failure to differentiate a recrudescence (same parasite strain) from a newly acquired infection (different parasite strain). A nested PCR analysis was conducted on paired dried blood spots (day 0 and day of treatment failure) for treatment outcomes classified as late treatment failures (LCF or LPF). PCR correction was made based on fragment analysis on gel electrophoresis. The WHO recommends the use of three markers (*msp1*, *msp2*, and *glurp*) were to make primary end point analysis. The current study used two of the three markers, the most polymorphic *msp2*, and the less polymorphic *msp1* (Additional file 2: Table S2 and Additional file 3: Table S3). The other highly polymorphic marker result was not included for technical and logistic reasons. PCR genotyping of the polymorphic genes was done at Ethiopian Public Health Institute (EPHI) according to the recommendation of the WHO.

### Ethical consideration

The study was initiated after getting ethical clearance from the institutional ethical review board of the Scientific and Ethical Review Office of EPHI and Ethics Review Committee of the School of Pharmacy, Addis Ababa University. Letter of Permission to conduct the study was also obtained from Arbaminch Zuria district office and Shecha health centre. Patients were enrolled in the study after informed consent (parents or guardians for children). Additional assent was obtained for children from age 12 to 17 years.

### Statistical analysis

Data entry and analysis were done using the WHO designed Excel spreadsheet. Efficacy data was analysed using the Kaplan-Meier (K-M) and per-protocol (PP) analysis methods. In PP analysis, the efficacy estimate (the proportion cured) of AL was derived by taking all participants followed until treatment failure and adequate response while excluding those who were withdrawn and lost to follow-up. In the K-M approach, patients without treatment failure during the study period and who did not complete follow-up due to withdrawal or lost to follow-up were included in the analysis until the last recorded visit when they were censored. In addition to the criteria for withdrawal, patients were withdrawn from the analysis when PCR results were unclassifiable.

## Results

### Study participants’ enrolment

**Fig. 1:**
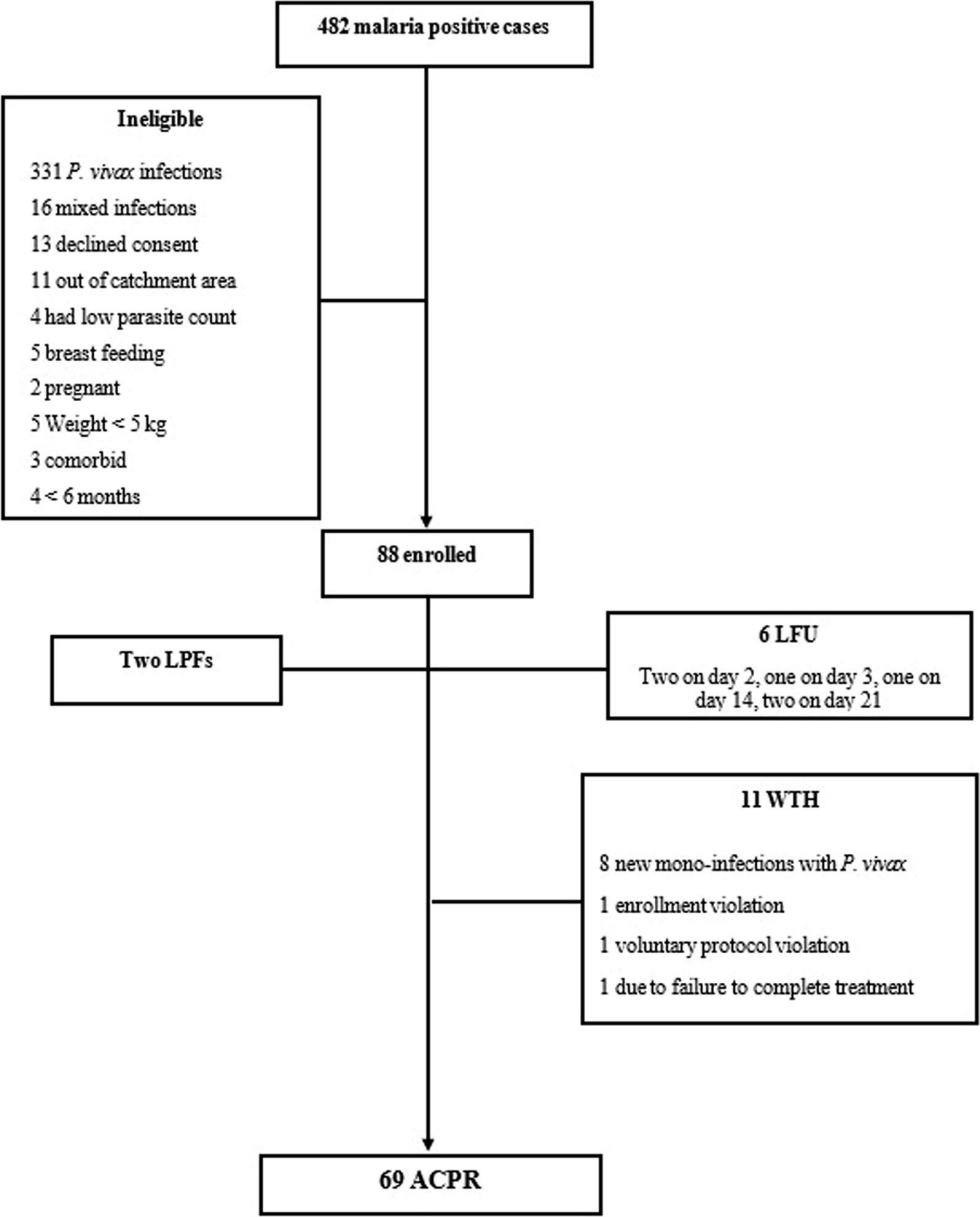
Flowchart depicting follow-up and outcomes of the study participants. *LFU*, Lost to follow-up; *WIH*, Withdrawal; *LPF*, Late parasitological failure; *ACPR*, Adequate clinical and parasitological response

A total of 3188 clinically suspected patients were screened for malaria. Of these 482 were microscopically found positive for malaria. Of the positive cases, 135 were *P. falciparum* infections, 331 were *Plasmodium vivax* infections, while 16 were mixed infections. A total of 88 consented patients who fulfilled the inclusion criteria were included in the study. Seventeen participants did not complete the study due to withdrawal and lost-to follow-up (Fig. 1).

### Baseline characteristics of the study participants

Among the total of 88 participants included in the study, 56 (63.6%) were males and 32 (36.4%) were females. Of these, 8 (9.1%) were under 5 years of age, 35 (39.8%) were in the age group 5–15 years, whereas 45 (51.1%) were adults (≥ 15 years). The mean age of the participants was 18.6 with a mean weight of 41.6 kg. The mean axillary body temperature, parasite count and haemoglobin level at baseline were 38.1 °C, 11,920/µl and 12.2 g/dl, respectively (Table 1).

**Table 1:** Baseline characteristics of study participants in the therapeutic efficacy study of artemether-lumefantrine for the treatment of uncomplicated *P. falciparum* malaria, Shecha health centre, Arba Minch, Ethiopia, 2021

Patient characteristics		Age group			
		(0–5)	[5–15)	≥ 15	Total
Mean age (range)		3.3 (2–4.9)	10.2 (5–14)	27.8 (15–73)	18.6 (2–73)
Gender					
Male n (%)		3 (37.5)	24 (68.6)	29 (64.4)	56 (63.6)
Female n (%)		5 (62.5)	11 (31.4)	16 (35.6)	32 (36.4)
Weight (kg), mean (SD)		14.1 (2.9)	29.4 (10.3)	56.1 (9.1)	41.6 (18.0)
Temperature (°C), mean (SD)		38.1 (1.3)	38.0 (1.2)	38.2 (1.3)	38.1 (1.2)
Parasitaemia (per µl), geometric mean (range)		17978.4 (3120–134125)	14809.5 (200–79160)	9359.7 (1000–76,440)	11920.5 (200–134125)
Gametocyte carriage n (%)		0 (0.0)	1 (2.9)	2 (4.4)	3 (3.4)
Haemoglobin (g/dl), mean (SD)		10.9 (1.6)	11.8 (1.8)	12.7 (1.9)	12.2 (1.9)

*SD* standard deviation, *kg* kilogram, *n* number, *°C* degree centigrade, *g/dl* gram/deciliter

### Treatment outcomes

#### Primary treatment outcomes

Sixty-nine of the participants completed the study with ACPR. Two LPFs, one on day 14 and the other on day 21, were observed. As per the per-protocol analysis, the PCR-uncorrected cure-rate of AL was 97.2% (95% CI 90.22–99.7%) (Table 2). According to the PCR-uncorrected Kaplan-Meier survival analysis, the cumulative incidence of success rate of AL was 97.5% (95% CI  90.5–99.4%).

**Table 2:** Artemether-lumefantrine efficacy endpoints in uncomplicated *P. falciparum* malaria, Shecha health centre, Arba Minch, Ethiopia, 2021

Efficacy endpoints	n [%]
ETF	0
LCF	0
LPF	2^**a**^
ACPR	69 [97.2%]
Total patients at baseline	88
PP PCR uncorrected cure rate	97.2% (95% CI 90.2–99.7%)
PP PCR corrected cure rate	98.6% (95% CI 92.3–100%)
K-M PCR uncorrected cure rate	97.5% (95% CI 90.5–99.4)
K-M PCR corrected cure rate	98.7% (95% CI 91.4–99.8)

**Fig. 2:**
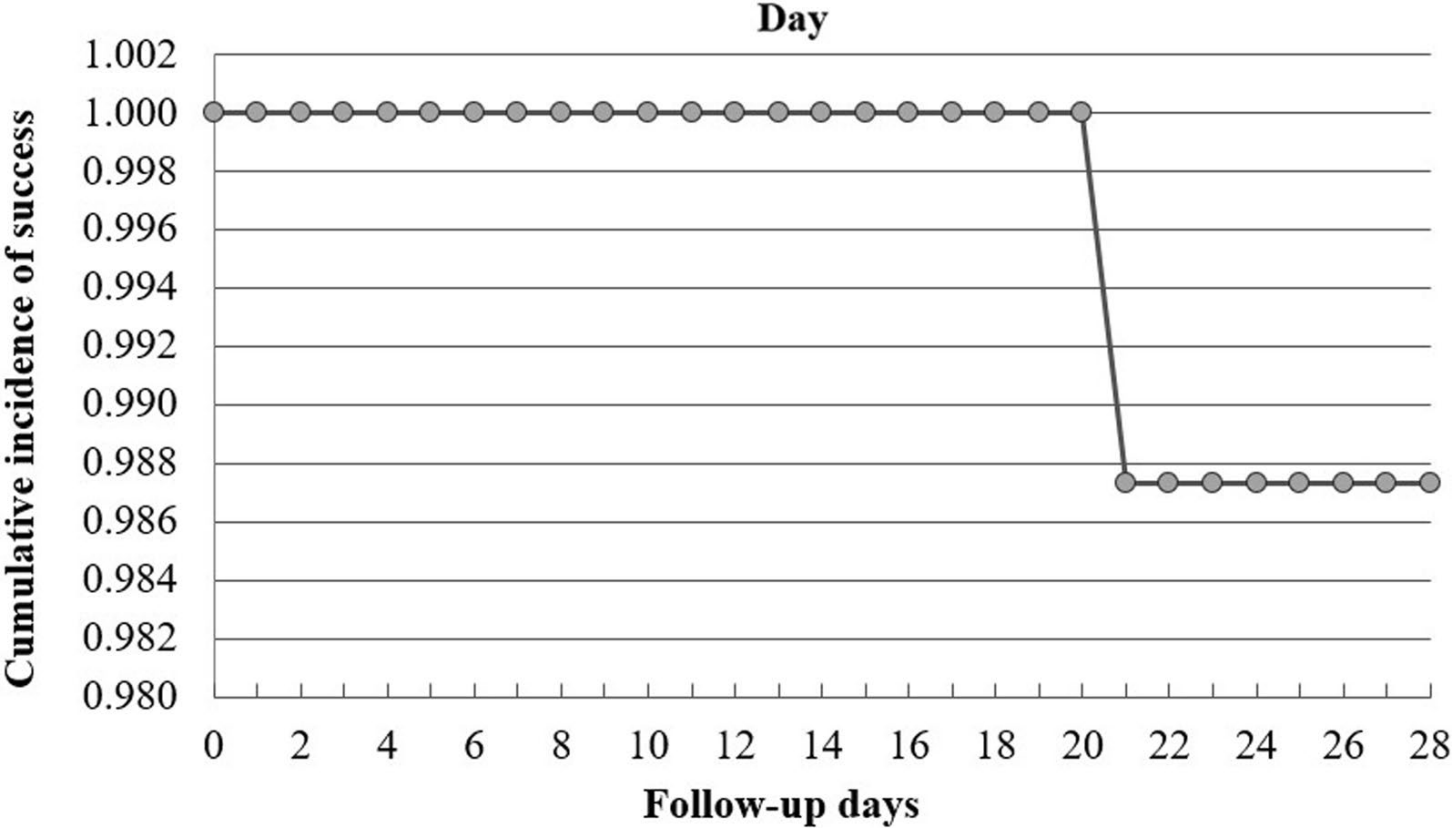
PCR corrected Kaplan–Meier Survival Curve

*n number, ETF* early treatment failure, *LCF* late clinical failure, *LPF* late parasitological failure, *ACPR* adequate clinical and parasitological response, *PP* per protocol analysis, *K-M* Kaplan-Meier, ^**a**,^one LPF was excluded with PCR correction,

When corrected with PCR, the LPF observed on day 21 was a recrudescence. The PCR results of the second LPF was unsatisfactory. Thus, it was classified as unknown and was excluded from the PCR-corrected analysis. As per the PCR-corrected per-protocol analysis, the cure rate of AL in this study was 98.6% (95% CI 92.3–100). The PCR-corrected Kaplan-Meier survival analysis, the cumulative incidence of success rate of AL was 98.7 (95% CI 91.4–99.8) (Fig. 2).

#### Secondary treatment outcomes

##### Parasite clearance

Based on the analysis of 83 of the participants (i.e., excluding one withdrawal on day one and two withdrawals and two LFUs on day 2) the day 3 positivity rate was 0.0% (95 CI 0.04.3).

##### Fever clearance

Out of the 88 participants, 57 (64.8%) were febrile (individuals with ≥ 37.5 ^o^ C axillary temperature) on the day of enrolment (day 0). The rest had a history of fever during the previous 24 h. Of the febrile patients on day 0, 39 (68.4%) were afebrile on day 1. On day 2, 52 (91.2%) of the participants were cleared of fever and 100% of the participants were cleared of fever on day 3. With this regard, the fever clearance rate of AL in this study was 68.4% on day 1, 91.2% on day 2 and 100% on day 3. No febrile cases were detected afterwards. The two treatment failures observed were afebrile on the day of presentation nor had a history of fever in the previous day.

##### Gametocyte carriage

On the day of enrolment, gametocytes were detected in three participants. Of the three gametocyte carriers on day 0, two of the participants cleared gametocytes on day 2 and the third case cleared gametocytes on day 3. No gametocyte carriage was observed afterwards. In this study, gametocyte carriage declined from 3.4% on day 0 to 1.2% on day 2 and to 0% on day 3.

##### Haemoglobin recovery

Haemoglobin was measured on day 0, day 14, and day 28. An increase in the mean haemoglobin level was observed from baseline to completion of follow-up. The mean haemoglobin on day 0 was 12.2 g/dl (Range: 8.3–16.2 g/dl) and was 12.3 g/dl (Range: 8.6–16.1 g/dl) on day 14. A slight increase in the mean haemoglobin was observed on day 28 to 12.7 g/dl (Range: 10-16.3 g/dl).

### Adverse events

Based on routine evaluation of worsening or new symptoms following treatment initiation, a total of 33 adverse events (AEs) were reported by 21 (23.9%) of the participants. Of the reported AEs, headache (8%), nausea (6.8%), anorexia (5.7%) and vomiting (4.5%) were the most common. Most of the AEs were reported in the first week of follow-up (day 0–day 3, and day 7) (Table 3). Except for diarrhoea (3.4%), for which rescue treatment was considered, all of the reported AEs resolved without sequel and rescue treatment. No serious adverse events were reported during the 28 day follow-up.

**Table 3:** Adverse drug events observed during the 28-day follow-up

AE	Follow-up days and Number (Frequency*) of AEs (N = 88), n (%)								Total n (%)
	Day-0	Day-1	Day-2	Day-3	Day-7	Day-14	Day-21	Day-28	
Headache	–	5 (5.7)	2 (2.3)	–	–	–	–	–	7 (8)
Anorexia	–	3 (3.4)	2 (2.3)	–	–	–	–	–	5 (5.7)
Nausea	2 (2.3)	4 (4.5)	–	–	–	–	–	–	6 (6.8)
Vomiting	2 (2.3)	2 (2.3)	–	–	–	–	–	–	4 (4.5)
Abdominal pain	–	–	–	1 (1.1)	–	–	–	–	1 (1.1)
Diarrhoea	–	–	1 (1.1)	1 (1.1)	1 (1.1)	–	–	–	3 (3.4)
Cough	–	–	1 (1.1)	1 (1.1)	1 (1.1)	–	–	–	3 (3.4)
Dizziness	–	–	–	1 (1.1)	1 (1.1)	–	–	–	2 (2.3)
Joint pain	–	–	–	–	–	1 (1.1)	–	–	1 (1.1)
Itching	–	–	–	–	–	–	1 (1.1)	–	1 (1.1)
Total n (%)	4 (4.5)	14 (15.9)	6 (6.8)	4 (4.5)	3 (3.4)	1 (1.1)	1 (1.1)	−	33 (37.5)

*AE* adverse event, *35.1% percent of the AEs were reported in the first follow-up week

### Clinical cases with treatment failure

According to the WHO recommendations, a result is classified as recrudescence if both paired samples have at least one identical band at both loci amplified. The distance migrated by fragments were visually inspected to verify recrudescence versus re-infection between day zero and day of failure samples for each marker (pretreatment and post treatment samples). Fragments with matching migrated distance were considered recrudescence. Accordingly, one of the treatment failures in the present study was classified as recrudescence. However, no amplification product was obtained for the second treatment failure. As the two markers gave no amplification product, PCR for *Plasmodium* species was performed, and no other *Plasmodium* species was identified. The blood film from the day of treatment failure was rechecked microscopically, which showed no *Plasmodium* species. Therefore, the result was interpreted as “unknown” and was excluded from the PCR-corrected analysis. This lack of PCR product could have been due to poor quality samples, insufficient DNA extraction, or both. It could also mean a false positive microscopy on the day of treatment failure. Cases of treatment failure were managed according to the national malaria treatment guideline of Ethiopia during the study period with quinine tablets (8.3 mg base/kg (= 10 mg quinine sulfate salt/kg) three times daily for seven days).

## Discussion

The present study demonstrates high efficacy of AL against uncomplicated *P. falciparum* in the study area. The PCR-corrected cure-rate of AL in our study is consistent with the PCR-corrected findings of previous studies conducted in Ethiopia [7, 10–15] and with recent studies elsewhere in Africa [16–18] after 28 days of follow-up period. Moreover, a recent review has also showed the high cure rate of AL in Ethiopia [19]. These results strongly support that AL remains highly efficacious two decades after its implementation.

No ETF or parasitaemia was detected on day 3. However, one LPF was classified as recrudescent malaria after PCR correction. In addition, AL also demonstrated a swift fever and gametocyte clearance, with no febrile cases or gametocyte carriage on day 3 and afterwards. The present study also revealed the favourable safety and tolerability profile of AL.

In Africa, despite detection of mutations in the *pfk13* gene, ACT still appear efficacious [20]. In this regard, the recrudescent case can be explained by the two factors that are considered as important determinants of AL treatment failure: host immunity and pharmacokinetic factors. Host-parasite interactions are primarily determined by the development of acquired immunity to malaria. Background immunity to malaria is believed to play a significant role in the efficacy of anti-malarials and is indeed associated with a lower risk of treatment failure [21–24]. It is believed to occur where malaria transmission is intense and develops in an age-dependent manner [25]. However, in areas where transmission is unstable, like Ethiopia, the development of premunition is unlikely [26].

According to a review, which did not detect the effect of fat, factors associated with lower day 7 lumefantrine concentration, a key factor for AL efficacy, were unsupervised (including partially supervised) treatment, younger children (≤ 4 years), and fever on admission [27]. Hence, the absence of host immunity and pharmacokinetic factors (which might have been influenced by partially supervised nature of this study and the participant being febrile on day 0), might play a role for the recrudescent case observed in this study. However, the reported recrudescence warrants further follow up TES studies supported by advanced molecular methods (amplicon sequencing).

In a TES, persistence of parasitaemia on day 3 after treatment initiation is interpreted as suspected artemisinin partial resistance. A 3 to 5% day 3 parasitaemia is expected in areas where parasites are fully sensitive [4]. In the present study, AL cleared parasitaemia as well as fever within 3 days. This has also been demonstrated with previously conducted studies in other parts of Ethiopia [13, 15, 28–30]. This explains the ability of artemisinins to swiftly clear parasites, resulting in a rapid resolution of clinical manifestations [31]. Other studies, however, detected parasitaemia and fever on day 3 [7, 12, 32–34] that may rather have been due to high levels of baseline parasitaemia [4]. In Africa, recent reports have showed evidence of slow-clearing *P. falciparum* infections [35–37]. A report from Rwanda provided the first evidence of artemisinin partial resistance [38]. These findings point to the possibility of clinically significant artemisinin resistance on the continent.

Artemisinin derivatives also have a gametocidal effect on sexual stages of *P. falciparum* [31]. This has been demonstrated in our study with clearance of gametocytemia on day 3. Similar findings have also been found in other Ethiopian studies [7, 14]. Several other studies, on the other hand, detected gametocytes on day 3 and afterwards [10–13, 15, 28–30]. This pattern of results is not surprising, given that the restricted or limited nature of ACTs gametocytocidal effect on immature gametocytes combined with the influence of other factors, results in the persistence of gametocytes for an average of more than one month after clearance of asexual parasites, posing a risk of residual transmission following complete clearance of asexual parasites [39–41]. Nonetheless, their wide-scale deployment has been significantly associated with a reduction in disease burden across a range of malaria endemic areas, including Ethiopia [42, 43].

The wealth of safety data on AL supports the favourable safety and tolerability profile of the drug. Most of the reported AEs are of mild or moderate severity and share a commonality with malaria or with concomitant infections [44–47]. Despite the differences in approaches to the assessment methods of prior Ethiopian studies on safety endpoints, except for a report of two severe adverse events in one study [10], none has reported a severe adverse event since the introduction of AL. The results of the present study provide supporting evidence that no serious adverse events were experienced and most of the reported events resolved with the clearance of parasitaemia and required no rescue treatment, suggesting that AL was tolerated by the participants during the 28 day follow-up period.

## Limitations

The study has the following limitations. The partial supervision of the study was a potential limitation. For logistical reasons, the second dose of AL was administered at home and verbally confirmed the next day. Another limitation of this study was the absence of day seven blood lumefantrine level measurement and advanced molecular marker analysis.

## Conclusion

The results of this study provide supporting evidence that AL remains highly efficacious two decades after its introduction in Ethiopia. High efficacy of AL in the treatment of uncomplicated *P. falciparum* malaria with rapid fever, parasite and gametocyte clearance in the study area was documented. Accompanying any future TES with advanced molecular analysis and pharmacokinetic data would help to better explain treatment failures and early detection of emerging and spreading resistance.

## Supplementary Information

Additional file 1. Table S1. Drug dosing and regimens.

Additional file 2. Table S2. Specific primers for msp1 and msp2.

Additional file 3. Table S3. Summary of genotyping of parasites.

## Data Availability

All data are available from the authors upon request.

## Abbreviations

AL: artemetherlumefantrine
WHO: World Health Organization
PCR: polymerase chain reaction
CI: Confidence interval
ACT: artemisinin-based combination therapy
TES: therapeutic efficacy studies
WBCs: white blood cells
ETF: early treatment failure
LCF: late clinical failure
LPF: late parasitological failure
ACPR: adequate clinical and parasitological response
EPHI: Ethiopian Public Health Institute
