# Supplementary material for: Safety and therapeutic efficacy of artemether-lumefantrine in the treatment of uncomplicated Plasmodium falciparum malaria at Shecha health centre, Arba Minch, Ethiopia
Source: Malar J. 2023 Jan 7;22:9. doi: 10.1186/s12936-022-04436-8 (PMC9824982; doi:10.1186/s12936-022-04436-8)
Supplement: Supplementary file 1 — Additional file 1. Table S1. Drug dosing and regimens. [file 12936_2022_4436_MOESM1_ESM.docx]

**Table S1. Drug dosing and regimens**

All patients were weighed to determine the accurate weight-based dose for the drug.

Artemether-lumefantrine (Coartem; Ipca Laboratories Ltd.) was administered twice daily for three days as tablets containing 20 mg of artemether plus 120 mg of lumefantrine in a fixed dose combination.

| **Weight (kg)** | **Day 0** | | **Day 1** | | **Day 2** | |
| --- | --- | --- | --- | --- | --- | --- |
|  | Morning | Evening | Morning | Evening | Morning | Evening |
| 5 to < 15 | 1 | 1 | 1 | 1 | 1 | 1 |
| 15 to < 25 | 2 | 2 | 2 | 2 | 2 | 2 |
| 25 to < 35 | 3 | 3 | 3 | 3 | 3 | 3 |
| ≥ 35 | 4 | 4 | 4 | 4 | 4 | 4 |
