# Supplementary material for: Safety and therapeutic efficacy of artemether-lumefantrine in the treatment of uncomplicated Plasmodium falciparum malaria at Shecha health centre, Arba Minch, Ethiopia
Source: Malar J. 2023 Jan 7;22:9. doi: 10.1186/s12936-022-04436-8 (PMC9824982; doi:10.1186/s12936-022-04436-8)
Supplement: Supplementary file 2 — Additional file 2. Table S2. Specific primers for msp1 and msp2. [file 12936_2022_4436_MOESM2_ESM.docx]

**Table S2. Specific primers for msp1 and msp2**

| **Locus** | **Allele** | **Primer** | | **Sequence (5′ to 3′)** |
| --- | --- | --- | --- | --- |
|  |  | **Forward** | **Reverse** |  |
| *msp1* | N/A | **🗸** |  | CTAGAAGCTTTAGAAGATGCAGTATTG |
|  |  |  | **🗸** | CTTAAATAGTATTCTAATTCAAGTGGACTA |
|  | K1 | **🗸** |  | AAATGAAGAAGAAATTACTACAAAAGGTGC |
|  |  |  | **🗸** | GCTTGCATCAGCTGGAGGGCTTGCACCAGA |
|  | MAD20 | **🗸** |  | AAATGAAGGAACAAGTGGAACAGCTGTTAC |
|  |  |  | **🗸** | ATCTGAAGGATTTGTACGTCTTGAATTACC |
|  | RO33 | **🗸** |  | TAAAGGATGGAGCAAATACTCAAGTTGTTG |
|  |  |  | **🗸** | CATCTGAAGGATTTGCAGCACCTGGAGATC |
| *msp2* | N/A | **🗸** |  | ATGAAGGTAATTAAAACATTGTCTATTATA |
|  |  |  | **🗸** | ATATGGCAAAAGATAAAACAAGTGTTGCTG |
|  | FC27 | **🗸** |  | GCAAATGAAGGTTCTAATACTAATAG |
|  |  |  | **🗸** | GCTTTGGGTCCTTCTTCAGTTGATTC |
|  | 3D7/IC | **🗸** |  | AGAAGTATGGCAGAAAGTAAKCCTYCTACT |
|  |  |  | **🗸** | GATTGTAATTCGGGGGATTCAGTTTGTTCG |
