# Supplementary material for: Safety and therapeutic efficacy of artemether-lumefantrine in the treatment of uncomplicated Plasmodium falciparum malaria at Shecha health centre, Arba Minch, Ethiopia
Source: Malar J. 2023 Jan 7;22:9. doi: 10.1186/s12936-022-04436-8 (PMC9824982; doi:10.1186/s12936-022-04436-8)
Supplement: Supplementary file 3 — Additional file 3. Table S3. Summary of genotyping of parasites. [file 12936_2022_4436_MOESM3_ESM.docx]

**Table S3. Summary of genotyping of parasites**

| **No^a^.** | **Sample ID** | **Day of recurrence** | **Parasitemia at day of recurrence** | **Type of failure** | **PCR result**  **(*msp1 & msp2*)** |
| --- | --- | --- | --- | --- | --- |
| 1 | 012 | 21 | 3160 (vs 8920 at day 0) | LPF | Recrudescence |
| 2 | 047 | 14 | 4320 (vs 3120 at day 0) | LPF | Negative**^a^** |

**^a^**one of the treatment failures, for which microscopy was positive, found negative with DNA amplification (PCR correction), probably due to either false positive microscopy or low-quality DNA.
